# Peritoneal dialysis catheter complications in neonates with kidney failure

**DOI:** 10.1007/s00467-026-07323-5

**Published:** 2026-05-01

**Authors:** Shannon Reinert, Maanasa Yarlagadda, Alexander Bondoc, Donna Claes, Stefanie Riddle, Meredith P. Schuh

**Affiliations:** 1 Division of Nephrology and Hypertension, Cincinnati Children’s Hospital Medical Center, Cincinnati, OH USA; 2 Division of Pediatric General and Thoracic Surgery, Cincinnati Children’s Hospital Medical Center, Cincinnati, OH USA; 3 Department of Surgery, University of Cincinnati College of Medicine, Cincinnati, OH USA; 4 Division of Pediatric Nephrology, Children’s Mercy Hospital, Kansas City, MO USA; 5 Department of Pediatrics, University of Missouri-Kansas City College of Medicine, Kansas City, MO USA; 6 Division of Neonatology, Perinatal Institute, Cincinnati Children’s Hospital Medical Center, Cincinnati, OH USA; 7 Department of Pediatrics, University of Cincinnati College of Medicine, Cincinnati, OH USA; 8 Division of Developmental Biology, Cincinnati Children’s Hospital Medical Center, Cincinnati, OH USA

**Keywords:** Neonatal kidney failure, Peritoneal dialysis, Pediatric dialysis

## Abstract

**Background:**

Neonates with kidney failure require placement of a peritoneal dialysis catheter (PD–C) shortly after birth. PD–C malfunction and peritonitis, both common in this population, are associated with PD failure. The aim of this study was to describe our center’s PD–C-related practices and outcomes in neonates with kidney failure.

**Methods:**

We performed a retrospective chart review of neonates who required kidney replacement therapy (KRT) within the first 30 days of life from 01/2018–12/2023. We evaluated for PD–C duration, PD–C replacement or revision, and peritonitis.

**Results:**

Twenty-four neonates had PD–C placed for chronic dialysis at a median of 9.7 days and 3 kg. Peritoneal dialysis (PD) was initiated a median of 14 days after PD–C placement, at a median age of 26.5 days. During a median follow-up time of 26.2 months, there were 42 PD–Cs tracked. Initial PD–C lasted a median of 355 days (IQR 165–632, range 50–953). Reasons for removal of initial PD–C included mechanical complications (38%), kidney transplant (25%), and infection (21%). There were 44 episodes of peritonitis in 19 patients. Seventeen of these episodes (39%) occurred within four weeks of a surgical procedure, despite all patients but one receiving perioperative antibiotic prophylaxis for all surgical procedures. The overall rate of peritonitis was 0.34 per 100-catheter days.

**Conclusions:**

PD–C complications are common in this highly complex population, despite mitigation attempts with perioperative antibiotics. Future work should focus on neonatal-specific recommendations for surgery timings and prophylaxis to reduce the risk of infection and PD failure in the neonatal kidney failure population.

**Graphical abstract:**

**Figure:**
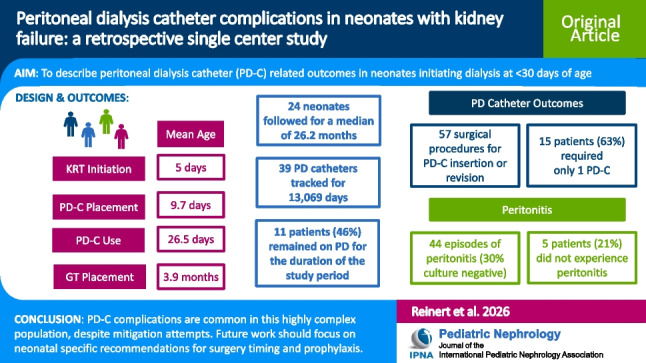
A higher resolution version of the Graphical abstract is available as Supplementary information

**Supplementary Information:**

The online version contains supplementary material available at 10.1007/s00467-026-07323-5.

## Introduction

Neonatal kidney failure is a rare occurrence, with a 2007 study reporting an incidence of 0.32 per 100,000 live births in the United States and Canada [1]. Anhydramnios due to fetal anuria was previously fatal due to severe pulmonary hypoplasia [2]. However, serial amnioinfusions in pregnancy can allow for adequate pulmonary development in these patients, increasing the number of surviving neonates born with kidney failure. Thus, with advances in fetal interventions, this population has been increasing in volume in recent years.

Peritoneal dialysis (PD) is the preferred dialysis modality in children [3]. Advantages include preservation of residual kidney function and vascular access, fewer dietary restrictions, no need for systemic anticoagulation, and the ability to perform dialysis at home. The most common complications of PD in pediatric patients are catheter malfunction and peritonitis, both of which are associated with increased risk of PD failure [4]. Age less than 1 year has repeatedly been demonstrated to be an independent risk factor for development of PD-associated peritonitis [5], need for peritoneal dialysis catheter (PD–C) revision [6], and PD–C leakage [6]. Small size at time of PD–C placement (< 12.4 kg) is also a risk factor for PD–C leakage [7]. The presence of a gastrostomy tube (GT) is a known risk factor for PD-associated peritonitis in pediatric patients [6, 8, 9], yet many patients with neonatal kidney failure require a GT to provide adequate nutrition [10]. While it is recommended for GT to be placed before or concurrently with PD–C to decrease risk of peritonitis [9, 11], this is not always feasible in this population due to the need to initiate kidney replacement therapy (KRT) shortly after birth due to anuria or oliguria. Finally, peritonitis in infants receiving PD has been associated with increased mortality and longer duration of initial hospitalization [9]. Thus, this neonatal kidney failure population is at increased risk for morbidity and associated mortality due to their young age and low weight.

Although there have been several studies investigating risk factors for PD–C-related complications in children, limited data exist regarding the risk of PD–C complications specifically in neonates with kidney failure. The small number of neonates with kidney failure has limited the ability to study outcomes in this population. The recently published COINED study reported on initial modality choice, modality changes, and mortality rates in neonates starting dialysis at ≤ 30 days of age, including 119 neonates with stage 5 chronic kidney disease [12]. However, this study did not evaluate for PD-related complications (e.g., peritonitis, PD–C malfunction) or outcomes after initial discharge. One large study evaluated peritonitis rates and patient outcomes in 156 infants, from 29 different centers, who had a PD–C placed at less than one year of age, including 83 patients with a PD–C placed at less than 30 days of age [9]. Patients who developed peritonitis were significantly younger and more likely to have had the PD–C placed at less than 30 days of age. The same study reported that GT insertion after PD–C placement resulted in a nearly threefold increased risk of peritonitis. However, this study included a heterogeneous patient population, with only 61% of patients having CAKUT. Limited data exist regarding timing of GT placement and associated outcomes in neonates initiating KRT within the first 30 days of life. This population is often unable to follow current guidelines regarding timing of GT placement due to the timing of and size at KRT initiation.

The aim of this study was to describe PD–C-related outcomes in this highly unique and complex population and identify any potentially related factors that should be the focus of future studies.

## Methods

We conducted a retrospective analysis of neonates with kidney failure undergoing PD–C placement at Cincinnati Children’s Hospital Medical Center (CCHMC) who were born between January 1, 2018, and December 31, 2023, and initiated KRT within 30 days of life. Patients receiving dialysis for acute kidney injury were not included. Institutional Review Board approval was obtained prior to record retrieval (approval no. 2023-0235 and 2024-0158). 

We identified 26 neonates who initiated KRT for kidney failure within the first 30 days of life. One patient was excluded because the PD–C was placed at an outside hospital. A second patient was excluded because goals of care were redirected to comfort care, and the PD–C was not utilized, leaving 24 patients that met study criteria. Our center follows International Society for Peritoneal Dialysis (ISPD) guidelines with placement of PD–C, including a downward-facing exit site, performing omentectomy, and providing perioperative antibiotics [11].

PD catheters placed in this patient cohort during the study period were placed surgically in the same manner: inserted through the rectus sheath and fixed in place with a fine permanent suture. The catheter was then tunneled subcutaneously superiorly to the umbilicus and through a downward-facing exit site which was then dressed with a chlorhexidine-impregnated disc and occlusive dressing. A partial or near-total omentectomy was also performed at the same time. Baseline demographic characteristics were collected for the 24 neonates in the study population. We evaluated for the following outcomes: episodes of peritonitis, relationship between peritonitis episodes and surgical procedure, PD–C revisions, PD–C removal, and duration of initial hospitalization. Diagnosis of peritonitis was made based on the ISPD guidelines [11]. Peritonitis in which no causative organism is identified is classified as culture negative peritonitis. Peritoneal effluent collection was performed as detailed in a process improvement study previously published by our center [13].

For the cohort of patients followed from PD–C placement through kidney transplant, age at transplant and modality at time of transplant were also assessed. Follow-up time was defined as time from birth to date of transfer out of institution, date of transplantation, or date at time of analysis, whichever was first. Patients who transferred institutions with their initial PD–C in place were censored at the time of transfer, with regard to the calculation of initial PD–C duration. Patients were considered to have received perioperative antibiotics if they received at least one dose of intraoperative antibiotics. A peritonitis episode was considered related to GT placement or PD–C placement/revision if it occurred within four weeks of the procedure. Short-term transitions to hemodialysis (HD for recovery after intra-abdominal surgeries) were not counted as transitioning to HD. Descriptive statistics performed included median values and interquartile ranges (IQR) or frequencies and proportions as appropriate using Excel. Given the limited sample size, additional statistical analysis was not performed.

### Center specific protocols

For continuity, our center has a designated group of kidney transplant surgeons who place PD–C, HD lines, and GTs. Patients must be at least 1.8 kg to be considered a chronic dialysis candidate. There is not necessarily a size cut off for PD–C placement, but rather a gestational age. If a patient is premature, the skin and abdominal wall are often underdeveloped or fragile, and a PD catheter cuff may not adequately be covered or may erode. Based on surgical assessment of the patient and their abdominal wall musculature and skin morphology, an HD catheter alone may be initially placed, with PD–C placement at a later date. We were not able to obtain data related to PD–C cuff number or length from the electronic medical record (EMR). Our current practice is to use a coiled, double-cuffed catheter based on our center’s history. We briefly trialed using a straight catheter based on adult data demonstrating decreased mechanical complications with a straight catheter [14, 15]. However, we noted increased need for revision/replacement and returned to using coiled PD–C. Our preferred exit site is the right lateral abdomen, inferior to the costal margin. While this can approach the Gibson incision for future transplant, we prefer to keep the site contralateral to the future gastrostomy site and any possible leakage inferiorly onto the catheter. As such, per the ISPD guidelines, the entrance site is transabdominal via the left rectus muscle, with a supraumbilical tunnel leading to a downward-facing exit.

PD is felt to be the ideal modality for this patient population to minimize blood exposure associated with continuous kidney replacement therapy (CKRT), provide continuous dialysis (with optimized fluid status and electrolytes), and provide a more holistic approach to daily care including increased holding time for families. Therefore, a PD–C is placed as soon as the infant is of appropriate size and the morphology of the abdominal wall is mature enough. An HD line is typically placed concomitantly with PD–C, so CKRT can be utilized immediately to prevent fluid overload and allow for adequate nutrition for improved development and wound healing while the PD–C heals. Our preferred method of feeding access is a primary gastrostomy button. Given that the smallest French size available is 12 Fr with a 2.5 ml luminal retention balloon, we typically allow for adequate growth of the infant and their gastric lumen via oral and/or nasogastric tube intake. If the infant has successfully transitioned to PD, but still has an HD catheter in place, we elect to return to the operating room for laparoscopic-assisted gastrostomy button placement around 2 months of post-natal age to place the GT. We allow for healing without PD to prevent infection and utilize just HD for 10–14 days. Our center uses CARPEDIEM, aquaphereis, and modified aquapheresis for extracorporeal support. While there is no standardized protocol for nutrition management, nutrition is generally liberalized during extracorporeal support to allow for optimized growth and wound healing while avoiding fluid overload. At the time of this study, there was no standardized protocol for perioperative antibiotic prophylaxis in patients with a PD–C; however, clinical pharmacists were involved in discussion of redosing after CKRT. The standard practice at our center is to hold PD for a minimum of 14 days after any PD–C revision or replacement.

Given our center’s experience with bilateral renal agenesis (BRA) and the poor outcomes associated with this diagnosis [16], our center does not currently offer prenatal interventions in this population. Therefore, there were no patients with BRA included in this study.

## Results

### Patient characteristics

Patient characteristics of the 24 neonates that met study criteria are summarized in Table 1. Fifteen patients (63%) were male. Underlying diagnoses included MCDK and/or renal agenesis (58%), bladder outlet obstruction (BOO, 38%), and cystic dysplasia (4%). All but two patients required prenatal interventions, consisting of vesicoamniotic shunting and/or amnioinfusions. Patients were followed for a median of 26.2 months [interquartile range (IQR) 18.1–31.6, range 2–67].

**Table 1:** Demographic and clinical characteristics of 24 neonates with kidney failure

Total subjects (M/F)	24 (15/9)	
Diagnosis, *n* (%)	Bladder outlet obstruction	9 (38%)
	Cystic dysplasia	1 (4%)
	MCKD/unilateral renal agenesis	5 (21%)
	Bilateral MCDK	9 (38%)
Mean age	Gestational age at birth,* weeks*	36.45 (range 31.7–38.3)
	At KRT initiation, *days*	5 (range 2–12)
	At PD–C placement, *days*	9.7 (range 1–46)
	At PD–C use, *days*	26.5 (range 9–59)
	At GT placement,* months*	3.9 (range 1.3–21.1)
Mean weight, *kg*	At Birth	2.6 (range 1.7–3.9)
	At PD–C placement	3 (range 2.1–4.5)
	At GT placement	4.8 (range 2.9–9.2)
Initial PD–C type (intra-abdominal segment), *n* (%)	Straight	4 (17%)
	Coiled	20 (83%)
Initial PD–C exit site, *n* (%)	Left abdomen	16 (67%)
	Mid abdomen	2 (8%)
	Right abdomen	6 (25%)
Omentectomy, *n* (%)	Total or partial omentectomy	20 (83%)
	Not Specified	3 (13%)
	None	1 (4%)
Median time between initial PD–C insertion and:	PD initiation, *days*	14 (IQR 13–17.25, range 6–32)
	GT placement*, days*	62 (IQR 49–106, range 0–641)
Median follow up time, *months*		26.2 (IQR 18.1–31.6, range 2–67)

*M* Male, *F* Female, *MCDK* Multicystic dysplastic kidney, *PD–C* Peritoneal dialysis catheter, *GT* Gastrostomy tube, *KRT *Kidney replacement therapy

On average, patients were 9.7 days old at PD–C placement (range 1–46). Thirty-nine PD–C were placed in 24 patients and were tracked for a total of 13,069 catheter days (Table 2). All patients received CKRT prior to PD initiation per our institutional practice for neonates with kidney failure. Most patients (83%) had a partial or total omentectomy documented in their initial PD catheter procedure note. In one patient, omentectomy was attempted; however, it was not successful given the inability to visualize the omentum. PD was started at a median of 14 days after PD–C placement (IQR 13–17.25, range 6–32), at a median age of 26.5 days (range 9–59). All but four patients in this study waited at least 14 days after PD–C insertion prior to PD initiation.

**Table 2:** Patient and peritoneal dialysis catheter outcomes

Catheter days tracked:						
Total	13,069					
Initial PD–C	10,151					
Median initial PD–C duration, *days*	355 (IQR 165–632, range 50–953)					
Number per patient, *n* (%)	PD–C revisions		PD–C replacement	PD–C revisions or replacements		
Zero	13 (54%)		15 (63%)	11 (46%)		
One	4 (17%)		4 (17%)	2 (8%)		
Two	7 (29%)		4 (17%)	6 (25%)		
Three			1 (4%)	2 (8%)		
≥ Four				3 (13%)		
Reason for procedure, *n* (%)	Initial PD–C removal (*n* = 24)	PD–C revision (*n* = 18)		Any PD–C removal (*n* = 34)		PD–C revision or removal (*n* = 52)
Mechanical failure	9 (38%)	18 (100%)		15 (44%)		33 (63%)
Transplant	6 (25%)			9 (26%)		9 (17%)
Infection	5 (21%)			9 (26%)		9 (17%)
Transferred with initial PD–C	3 (13%)					
Initial PD–C still in use	1 (4%)					
Transition to HD for social reasons				1 (3%)		1 (2%)
Median age at any PD–C removal^a^ or revision (days)	321 (IQR 106–484, range 32–1213)					
Median duration of initial hospitalization (days)	144 (IQR 103–181, range 60–1525)					
Modality at hospital discharge, *n* (%)						
Discharged home on PD	15 (63%)					
Transferred on PD	4 (17%)					
Discharged home on HD	3 (13%)					
Transferred on HD	1 (4%)					
Transplant	1 (4%)					
Cohort followed through transplant						
Total subjects	16					
Mean age at transplant, *years*	2.4 (range 1.2–3.9)					
Modality at transplant, *n* (%)	HD			7 (44%)		
	PD			9 (56%)		

^a^Only PD–C removed for mechanical failure or infection included

*PD–C*, peritoneal dialysis catheter; *PD*, peritoneal dialysis; *HD*, hemodialysis

Our program also standardizes placement of a GT in all neonates with kidney failure during the initial hospitalization prior to discharge. Given the need to initiate dialysis soon after birth, GT placement is often completed after PD–C placement. Thus, twenty-one of twenty-four patients had the GT placed after PD–C, as a single surgical procedure or concomitant with another surgical procedure. Two patients had delayed PD–C placement due to size at time of HD catheter placement, and had GT placed concurrently with PD–C placement. One patient was transferred to another institution at 60 days of life and had not yet had a GT placed. Patients were on average 3.9 months old at GT placement (range 1.3–21.1 months). 

### Patient and peritoneal dialysis catheter outcomes

Thirty-nine PD–C were placed in 24 patients, tracked for a total of 13,069 catheter days (Table 2). Median survival of the initial PD–C was 355 days (IQR 165–632, range 50–967), with three catheters censored at time of institutional transfer. Fifteen patients (63%) required only one PD–C; 11 of these patients also did not require any PD–C revision, whereas the remaining 4 patients required a total of seven revisions.

Reasons for removal of initial PD–C included mechanical failure (*n* = 9, 38%), kidney transplant (*n* = 6, 25%), and infection (*n* = 5, 21%). One initial PD–C was still in use at this institution at the time of study, and three patients had transferred to another institution with their initial PD–C. In total, there were 57 surgical procedures related to either PD–C insertion or revision (39 PD–C insertions plus 18 revisions). Mechanical failure was the most common reason for any PD–C removal (*n* = 15, 44%) and was the reason for all PD–C revisions (*n* = 18). Of the forty-two procedures (removal or revision) performed for infection or mechanical failure, 19 (45%) occurred during the initial hospitalization.

There were 44 episodes of peritonitis in 19 unique patients (Table 3). The overall rate of peritonitis was 0.34 per 100 catheter days. Twenty-two peritonitis episodes (50%) occurred in 13 unique patients during the first year of life. Of those 22 peritonitis episodes, the majority (15 peritonitis episodes in 10 unique patients) occurred during the initial hospitalization. Seventeen of the 44 peritonitis episodes (39%) occurred within 4 weeks of a surgical procedure. Only one patient did not receive antibiotic prophylaxis at the time of PD–C placement; otherwise, perioperative antibiotic prophylaxis was administered for every PD–C placement, PD–C revision, and GT placement. The majority of the time (53 of 76 procedures), cefazolin was the antibiotic used. Cefoxitin was used in 15 procedures (along with vancomycin in 2 procedures). In a small number of cases, ampicillin (*n* = 2), cefepime (*n* = 1), clindamycin (*n* = 1), or vancomycin (*n* = 4) were used, typically due to the patient already being on the antibiotic for another indication. Duration of antibiotic treatment ranged from 1 to 3 days. There was no trend between class and/or duration of antibiotic used and development of peritonitis within 6 weeks of procedure. Five patients (21%) did not experience peritonitis during the study period. Of these five patients, two transferred institutions on PD at 2 and 4.5 months of age, the other three remained on PD until transplantation. Most patients (50%) had 1–2 episodes of peritonitis. One patient had eight episodes of peritonitis. Gram positive organisms accounted for the greatest percentage of infections (*n* = 19, 43%; Table 3). Thirteen episodes (30%) were culture negative. One peritonitis episode (2%) was fungal and one (2%) was polymicrobial.

**Table 3:** Peritonitis-related outcomes

	Episodes	Patients with peritonitis^a^
Total, *n*	44	19 (79%)
During initial hospitalization, *n* (%)	15 (34%)	10 (42%)
Within 4 weeks of procedure, *n* (%)	17 (39%)	11 (46%)
Within 4 weeks of GT placement,* n* (%)	5 (11%)	5 (21%)
During first year of life, *n* (%)	22 (50%)	13 (54%)
During two years of life, *n* (%)	39 (89%)	19 (79%)
Episodes per patient, *n* (%)		
Zero		5 (21%)
One		10 (42%)
Two		2 (8%)
Three		3 (13%)
> Three		4 (17%)
Peritonitis type, *n* (%)^b^		
Gram positive		19 (43%)
Gram negative		10 (23%)
Culture negative		13 (30%)
Gram positive and gram negative		1 (2%)
Fungal		1 (2%)

^a^Denominator of 24 (total number of participants in study)

^b^Denominator of 44 (total number of peritonitis events)

**Fig. 1:**
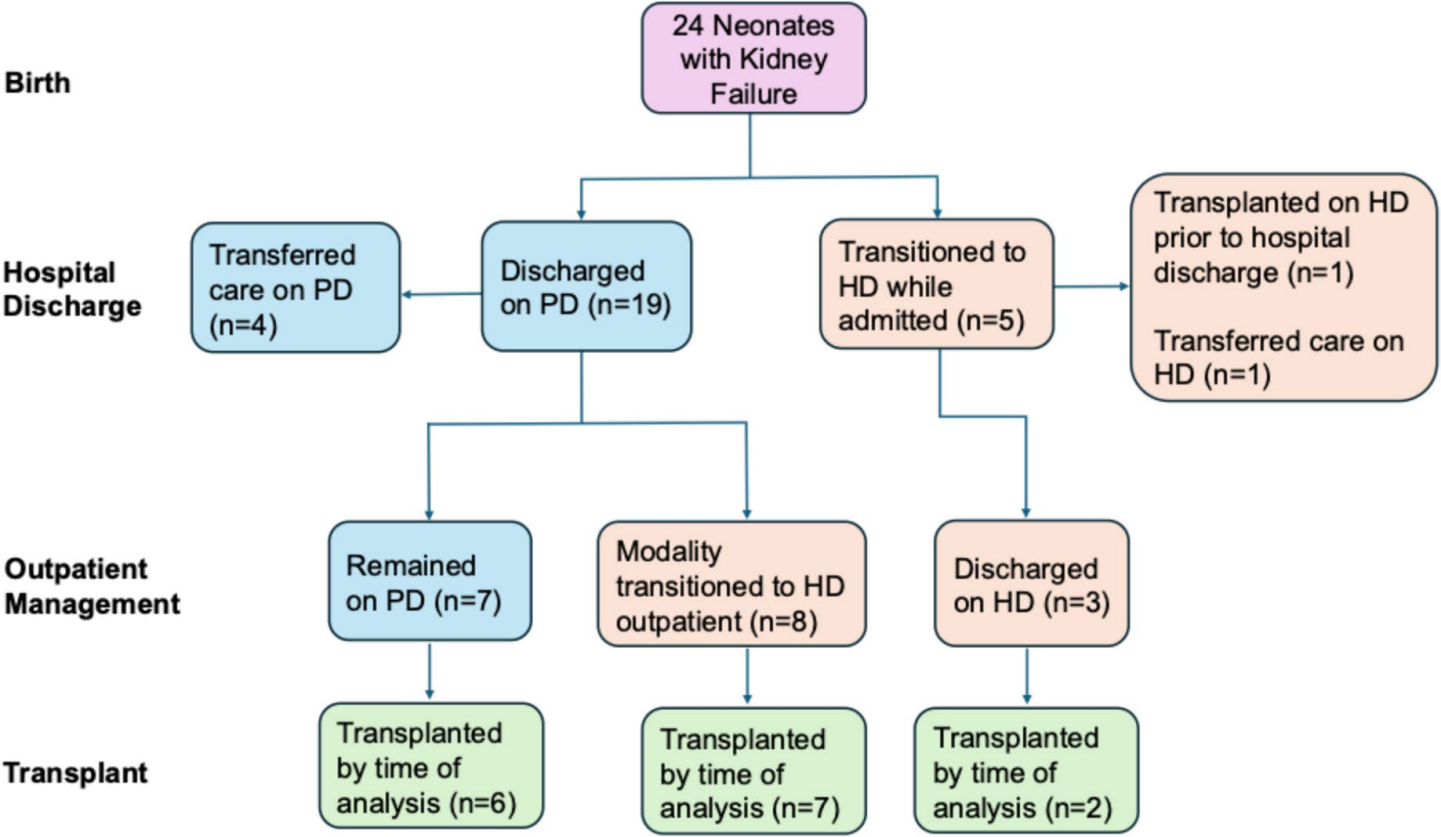
Flow chart demonstrating modality transitions while on dialysis. Reasons for transition to HD include mechanical failure (*n* = 8), recurrent/refractory peritonitis (*n* = 3), hepatoblastoma (*n* = 1), and social reasons (*n* = 1). *PD*, peritoneal dialysis; *HD*, hemodialysis

Median duration of initial hospitalization was 144 days (IQR 103–181, range 60–1525, Table 2). Modality outcomes and transitions are displayed in Fig. 1. Five neonates were transferred to other pediatric dialysis programs on PD (*n* = 4) or HD (*n* = 1) during the initial hospitalization. The remaining 19 patients maintained long-term dialysis management within our center. The dialysis modality at the time of discharge for these 19 patients were as follows: 15 on PD, 3 discharged to home on HD, and one remained admitted on HD until time of transplant due to medical complexity and social issues. Of the 15 discharged to home on PD, seven (47%) were able to be maintained on PD up until transplantation, whereas the other 8 patients (53%) transitioned from PD to HD. Two patients discharged to home on HD were able to transition back to PD for a period prior to transplantation. Of these 19 patients, 16 (84%) were transplanted during the study period at a mean age of 2.4 years (range 1.2–3.9). Modality at time of transplant: HD = 7, PD = 9. Three patients (median age 2.42 years; range 1.8–5.6 years) remain on dialysis therapy at our institutions (HD = 2, PD = 1; this PD patient is maintained on PD with his original PD–C). The survival rate was 100% at hospital discharge, and all patients still under the care of our center were alive at the time of study analysis. One patient passed away after transferring to another pediatric dialysis center, the other four transferred patients survived to transplant.

## Discussion

This study represents the challenges experienced by our single center regarding initiation and continuation of dialysis therapy in a cohort of neonates born with kidney failure and prenatal oligo- and anhydramnios. Despite having a 100% survival rate at hospital discharge, the burden to initiate and maintain PD was significant due to a combination of PD–C-associated surgical procedures (revisions and replacements) and peritonitis. Although approximately 60% of the cohort (*n* = 15) required only one PD–C, the remaining 40% (*n* = 9) required a total of 24 PD–C insertions. There was a high rate of peritonitis (especially during the initial hospitalization), with over a third of these peritonitis events being associated with a preceding surgical procedure. In addition, only 11 patients (46%) remained on PD for the duration of the study period. Thus, 54% (*n* = 13) of the cohort transitioned their long-term dialysis treatment modality from PD to HD, with five of these 13 patients transitioning to chronic HD therapy even prior to hospital discharge.

Aligned with the previous literature [6], our study demonstrates that PD–C complications are common, but that additional complexities arise with this unique and growing patient population. Prior studies have found several factors to be associated with increased risk of PD–C infection or malfunction, including young age (increased risk of reoperation for leak, hernia, and mortality at < 1 year of age, increased risk of infection at < 3 years of age, and increased risk of PD–C revision < 6 years of age) [6, 8, 17], concomitant GT [6, 8, 9], and early use of PD–C [8, 11, 18, 19]. These patients are inherently young and typically require a GT for adequate nutrition [10]. Although GTs should ideally be placed prior to or concurrently with PD–C placement, this is not always possible in neonates who are typically too small for GT placement at the time of PD–C placement. Only two out of twenty-four patients in this study were large enough for GT placement at the time of their PD–C placement. These patients had PD–C placed later in the admission based on surgeon preference at the time of HD catheter placement.

One complicating factor in the neonatal population is the need for urgent KRT. Providers must decide to either start PD earlier than the recommended 14 days following PD–C insertion or use CKRT as a bridge to PD. Although there is literature supporting waiting a minimum of three days between PD–C placement and use to reduce the risk of complications and improve catheter survival in the pediatric population [19], many centers instead chose to wait longer as allowing PD–C to heal for at least 14 days from time of placement decreases the risk of peritonitis in the first 30 days after catheter placement [18] as well as dialysate leak in adult patients [8]. All but four patients in this study waited at least 14 days after PD–C insertion prior to PD initiation. Concomitant omentectomy has been shown to be protective against PD–C malfunction [6, 20] and PD–C revision [17], by decreasing the chance of omentum wrapping around the PD–C, which leads to catheter occlusion. Yet, omentectomy was performed in all but four patients in our cohort. It should also be noted that even when an omentectomy is attempted, it is not always possible to perform complete omentectomy, as was the case for one patient in this study. In addition, administration of perioperative antibiotics has been shown to reduce the risk of infection post-operatively [21, 22], all but one of the surgical procedures in this study utilized perioperative antibiotics. Yet, despite following recommended guidelines as best as able (i.e., omentectomy with > 80% of PD–C insertions, administering perioperative antibiotics with all but one PD–C insertion, waiting 14 + days from the time of PD–C insertion to initiation of peritoneal dialysis in all but four patients, and downward exit-site orientation), mechanical failure, peritonitis, and need for PD–C revision or removal was still common. This raises the question of what other practices, diagnostic studies, and/or new technologies are needed to reduce the morbidity specific to the neonatal kidney failure population.

The rate of culture negative peritonitis was 30%, which is double the recommended benchmark of < 15% [11]. However, our rate was similar to reported rates of culture negative peritonitis rates in other large pediatric studies (27–29%) [23, 24]. One potential reason for high culture negative peritonitis rates is that the elevated white blood cell count in the peritoneal cavity may be due to inflammation and not infection. This puts patients at risk for unnecessary antibiotic use and is relevant to our study given that 39% of all peritonitis episodes in our cohort were within 4 weeks of any surgical procedure. However, other possible reasons for the high culture negative peritonitis rate are technique of peritoneal effluent collection, early peritonitis, and difficult to culture organisms [13, 23]. Standardization of PD fluid collection has previously been shown to significantly decrease rates of culture negative peritonitis. In addition, the standard clinical diagnosis of peritonitis relies on bacterial culture, which may lack sufficient sensitivity to detect early peritonitis and is easily affected by suboptimal dialysate sampling techniques [25]. To overcome some of the limitations of traditional bacterial cultures, some clinical laboratories are using molecular techniques. Research into the use of these types of diagnostic studies in the PD population is needed so that patients are appropriately treated.

Although we routinely administered prophylactic perioperative antibiotics, our center lacked a standardized antibiotic practice during the study years, in that the antibiotic chosen and duration of antibiotics varied from patient to patient. In addition, many patients received KRT immediately after their surgical procedure, which would have cleared their antibiotics. Although pre-operative antibiotics are recommended prior to PD–C insertion or for other surgical procedures in pediatric patients on PD, there are no recommendations regarding the duration of perioperative antibiotics, especially in the setting of CKRT or HD. We recommend that the relationship between duration of post-surgical antibiotic coverage and risk for infection should be evaluated in future studies.

While our center delays the start of PD–C via the use of extracorporeal circuits, this practice has its own associated risks [26], including exposure to blood primes, hypotension, HD catheter malfunction and risk of vascular occlusion and permanent loss of vascular access options—a significant complication that is unfortunately beyond the scope of this specific paper. As there are no published reports discussing the long-term risks of vascular access occlusion and loss of vascular access related to this practice in the neonatal population, and given that many pediatric centers are opting to use CKRT as a bridge to PD [12, 27], we recommend that future pediatric studies focus on reporting this finding and counseling families on this specific morbidity in addition to all of the morbidities associated with neonatal kidney failure. Despite these risks, the findings in this study do raise the question: would delay of PD–C placement via prolonged CKRT (with GT placement prior to or with PD placement) avoid some of these PD-specific complications discussed in this manuscript?

We also want to emphasize the finding that over half (54%) of all neonates in our cohort required transition to outpatient HD treatment. This is significantly higher than the rate of modality change reported by Vidal et al., which included 1063 infants requiring maintenance dialysis (25%) [3]. The majority of these modality switches in their study occurred after hospital discharge. In a recently published study by Muff-Luettt et al., only 16 of 119 (13%) patients with neonatal stage-5 chronic kidney disease (CKD-5) required a modality change for a dialysis complication during their initial admission. However, 50 patients (42%) died prior to discharge and after-discharge modality changes were not reported [12]. An additional notable difference is that of the 63 surviving patients who were discharged home on dialysis, only 57 (92%) were discharged home on PD. This is compared to the 79% who were discharged on PD in our study. In addition to the risks associated with having an HD catheter as previously mentioned, transitioning to HD puts a significant burden on families of small children, given that it typically means traveling to a dialysis unit 4–5 times a week, and many families live geographically far away from the nearest pediatric HD center. This demand for outpatient HD in small infants and children also has significant implications for both the number and skill required for the pediatric HD nursing workforce. Future studies should evaluate rates of modality transition in this population to determine if this high rate is replicated at other centers.

This study has important limitations. As a retrospective study, we were limited by the available data in the EMR including lack of granularity of all causes of catheter malfunctions and associated risks of peritonitis such as catheter leakage. In addition, our small sample size prevented us from doing any additional statistical analysis to evaluate for PD–C complication risk factors in this population. Despite these limitations, this study captures a previously understudied population of patients who initiated KRT within the first 30 days of life—with a median follow-up of 26.2 months, 13,069 catheter days tracked, and two-thirds of patients followed up until the time of kidney transplantation.

In conclusion, PD–C complications are common in this highly complex population, despite mitigation attempts with perioperative antibiotics, omentectomy, and delayed use of PD–C. While this study represents data only at our center, these same questions will be reflected at all centers now caring for neonates with kidney failure. Neonates with kidney failure require placement of a PD–C shortly after birth, and therefore the current ISPD-recommended guidelines regarding surgical timing cannot be followed. It is unclear if there are ways to mitigate the risk of peritonitis and PD failure in this population. Thus, we are in desperate need of research, new guidelines, and potential technologies focused on reducing PD–C complications and potential PD failure in the neonatal kidney failure population. Not only are these complications a significant burden to the patients and families, but the need to transition from PD to HD is a significant burden for the pediatric nephrology program as well. This study highlights the need for neonatal-specific recommendations for surgery timings and prophylaxis as this population continues to grow. Patients with diagnoses previously incompatible with life are now surviving, and our next steps must focus on reducing morbidities involving their complex care needs, including ways to mitigate risk of infection and PD failure in the neonatal kidney failure population.

## Supplementary Information

Below is the link to the electronic supplementary material.

ESM 1 Graphical abstract (PPTX 82.9 KB)

## Data Availability

The datasets generated and analyzed during the current study are available from the corresponding author on reasonable request.

## References

[1] Carey WA, Talley LI, Sehring SA, Jaskula JM, Mathias RS (2007) Outcomes of dialysis initiated during the neonatal period for treatment of end-stage renal disease: a North American Pediatric Renal Trials and Collaborative Studies special analysis. Pediatrics 119:e468-473 17224455 10.1542/peds.2006-1754

[2] Warring SK, Novoa V, Shazly S, Trinidad MC, Sas DJ, Schiltz B, Prieto M, Terzic A, Ruano R (2020) Serial amnioinfusion as regenerative therapy for pulmonary hypoplasia in fetuses with intrauterine renal failure or severe renal anomalies: systematic review and future perspectives. Mayo Clin Proc Innov Qual Outcomes 4:391–409 32793867 10.1016/j.mayocpiqo.2020.04.008 PMC7411166

[3] Vidal E, van Stralen KJ, Chesnaye NC, Bonthuis M, Holmberg C, Zurowska A, Trivelli A, Da Silva JEE, Herthelius M, Adams B, Bjerre A, Jankauskiene A, Miteva P, Emirova K, Bayazit AK, Mache CJ, Sánchez-Moreno A, Harambat J, Groothoff JW, Jager KJ, Schaefer F, Verrina E, ESPN/ERA-EDTA Registry (2017) Infants requiring maintenance dialysis: outcomes of hemodialysis and peritoneal dialysis. Am J Kidney Dis 69:617–625 27955924 10.1053/j.ajkd.2016.09.024

[4] Bonenkamp AA, van Eck van der Sluijs A, Dekker FW, Struijk DG, de Fijter CW, Vermeeren YM, van Ittersum FJ, Verhaar MC, van Jaarsveld BC, Abrahams AC (2023) Technique failure in peritoneal dialysis: modifiable causes and patient-specific risk factors. Perit Dial Int 43:73–83 35193426 10.1177/08968608221077461

[5] Chadha V, Schaefer FS, Warady BA (2010) Dialysis-associated peritonitis in children. Pediatr Nephrol 25:425–440 19190935 10.1007/s00467-008-1113-6 PMC2810362

[6] Phan J, Stanford S, Zaritsky JJ, DeUgarte DA (2013) Risk factors for morbidity and mortality in pediatric patients with peritoneal dialysis catheters. J Pediatr Surg 48:197–202 23331815 10.1016/j.jpedsurg.2012.10.035

[7] Stewart CL, Acker SN, Pyle LL, Kulungowski A, Cadnapaphornchai M, Bruny JL, Karrer F (2016) Factors associated with peritoneal dialysis catheter complications in children. J Pediatr Surg 51:159–162 26572851 10.1016/j.jpedsurg.2015.10.035

[8] Rahim KA, Seidel K, McDonald RA (2004) Risk factors for catheter-related complications in pediatric peritoneal dialysis. Pediatr Nephrol 19:1021–1028 15206031 10.1007/s00467-004-1520-2

[9] Zaritsky JJ, Hanevold C, Quigley R, Richardson T, Wong C, Ehrlich J, Lawlor J, Rodean J, Neu A, Warady BA, SCOPE Investigators (2018) Epidemiology of peritonitis following maintenance peritoneal dialysis catheter placement during infancy: a report of the SCOPE collaborative. Pediatr Nephrol 33:713–722 29150711 10.1007/s00467-017-3839-5

[10] Misurac J (2017) Chronic kidney disease in the neonate: etiologies, management, and outcomes. Semin Fetal Neonatal Med 22:98–103 27733241 10.1016/j.siny.2016.09.003

[11] Warady BA, Same R, Borzych-Duzalka D, Neu AM, El Mikati I, Mustafa RA, Begin B, Nourse P, Bakkaloglu SA, Chadha V, Cano F, Yap HK, Shen Q, Newland J, Verrina E, Wirtz AL, Smith V, Schaefer F (2024) Clinical practice guideline for the prevention and management of peritoneal dialysis associated infections in children: 2024 update. Perit Dial Int 44:303–364 39313225 10.1177/08968608241274096

[12] Muff-Luett M, Webb T, Scobell R, Pottanat N, Ciccia E, Claes D, Carter C, Nelson-Taylor S, Beebe M, Harris M, Alhamoud I, Joseph C, Grinsell M, Petgrave Y, Lande MB, Richardson K, Alzarka B, Razzouk R, Piburn K, Byfield R, Kim H, Katsoufis CP, Lindner C, Crawford B, Villegas L, High R, Lyden E, Sanderson K (2026) Dialysis modality and mortality of the Contemporary Infant and Neonatal Dialysis (COINED) Cohort: a Pediatric Nephrology Research Consortium (PNRC) study. Pediatr Nephrol. 10.1007/s00467-025-07082-9 41526758 10.1007/s00467-025-07082-9 PMC13139246

[13] Pangonis SF, Schaffzin JK, Claes D, Mortenson JE, Nehus E (2023) An initiative to improve effluent culture detection among pediatric patients undergoing peritoneal dialysis through process improvement. Pediatr Nephrol 38:211–218 35445978 10.1007/s00467-022-05533-1 PMC9021362

[14] Xie J, Kiryluk K, Ren H, Zhu P, Huang X, Shen P, Xu T, Chen X, Chen N (2011) Coiled versus straight peritoneal dialysis catheters: a randomized controlled trial and meta-analysis. Am J Kidney Dis 58:946–955 21872978 10.1053/j.ajkd.2011.06.026

[15] Chow KM, Wong SSM, Ng JKC, Cheng YL, Leung CB, Pang WF, Fung WWS, Szeto CC, Li PKT (2020) Straight versus coiled peritoneal dialysis catheters: a randomized controlled trial. Am J Kidney Dis 75:39–44 31445925 10.1053/j.ajkd.2019.05.024

[16] Riddle S, Habli M, Tabbah S, Lim FY, Minges M, Kingma P, Polzin W (2020) Contemporary outcomes of patients with isolated bilateral renal agenesis with and without fetal intervention. Fetal Diagn Ther 47:675–681 32516788 10.1159/000507700

[17] Schuh MP, Nehus E, Liu C, Ehlayel A, Clark S, Chishti A, Edwards-Richards AD, Erkan E, Jernigan S, Kamel M, Luckritz K, Magella B, Mansuri A, Wilson AC, Claes DJ (2021) Omentectomy reduces the need for peritoneal dialysis catheter revision in children: a study from the Pediatric Nephrology Research Consortium. Pediatr Nephrol 36:3953–3959 34128096 10.1007/s00467-021-05150-4

[18] Keswani M, Redpath Mahon AC, Richardson T, Rodean J, Couloures O, Martin A, Blaszak RT, Warady BA, Neu A, SCOPE Investigators (2019) Risk factors for early onset peritonitis: the SCOPE collaborative. Pediatr Nephrol 34:1387–1394 30969363 10.1007/s00467-019-04248-0

[19] Imani PD, Carpenter JL, Bell CS, Brandt ML, Braun MC, Swartz SJ (2018) Peritoneal dialysis catheter outcomes in infants initiating peritoneal dialysis for end-stage renal disease. BMC Nephrol 19:231 30217181 10.1186/s12882-018-1015-1 PMC6137733

[20] Lemoine C, Keswani M, Superina R (2019) Factors associated with early peritoneal dialysis catheter malfunction. J Pediatr Surg 54:1069–1075 30803792 10.1016/j.jpedsurg.2019.01.042

[21] Bender FH, Bernardini J, Piraino B (2006) Prevention of infectious complications in peritoneal dialysis: best demonstrated practices. Kidney Int Suppl. 10.1038/sj.ki.5001915 17080111 10.1038/sj.ki.5001915

[22] Sardegna KM, Beck AM, Strife CF (1998) Evaluation of perioperative antibiotics at the time of dialysis catheter placement. Pediatr Nephrol 12:149–152 9543378 10.1007/s004670050427

[23] Davis TK, Bryant KA, Rodean J, Richardson T, Selvarangan R, Qin X, Neu A, Warady BA (2021) Variability in culture-negative peritonitis rates in pediatric peritoneal dialysis programs in the United States. Clin J Am Soc Nephrol 16:233–240 33462084 10.2215/CJN.09190620 PMC7863662

[24] Schaefer F, Feneberg R, Aksu N, Donmez O, Sadikoglu B, Alexander SR, Mir S, Ha IS, Fischbach M, Simkova E, Watson AR, Möller K, von Baum H, Warady BA (2007) Worldwide variation of dialysis-associated peritonitis in children. Kidney Int 72:1374–1379 17882152 10.1038/sj.ki.5002523

[25] Ye J, Sun J, Chen D, Cao H, Lv Y, Ye C, Wang Y, Jiang H (2025) Culture-negative peritonitis and the latest diagnostic techniques. Kidney Dis (Basel) 11:25–37 40606677 10.1159/000542870 PMC12215107

[26] Battista J, De Luca D, Eleni Dit Trolli S, Allard L, Bacchetta J, Bouhamri N, Enoch C, Faudeux C, Guichoux J, Javouhey E, Kolev K, Regiroli G, Ranchin B, Bernardor J (2023) CARPEDIEM® for continuous kidney replacement therapy in neonates and small infants: A French multicenter retrospective study. Pediatr Nephrol 38:2827–2837 36625933 10.1007/s00467-022-05871-0

[27] Sanderson KR, Shih WV, Warady BA, Claes DJ (2024) Severe fetal CAKUT (congenital anomalies of the kidneys and urinary tract), prenatal consultations, and initiation of neonatal dialysis. Am J Perinatol 41:e156–e162 35554891 10.1055/a-1850-4429 PMC9734282

